# Antagonism of Interleukin-17A ameliorates experimental hepatic fibrosis by restoring the IL-10/STAT3-suppressed autophagy in hepatocytes

**DOI:** 10.18632/oncotarget.14266

**Published:** 2016-12-27

**Authors:** Xiao-Wei Zhang, Su Mi, Zhe Li, Ji-Chao Zhou, Jing Xie, Fang Hua, Ke Li, Bing Cui, Xiao-Xi Lv, Jiao-Jiao Yu, Zhuo-Wei Hu

**Affiliations:** ^1^ Molecular Immunology and Pharmacology Group, State Key Laboratory of Bioactive Substances and Functions of Natural Medicines, Institute of Materia Medica, Chinese Academy of Medical Sciences & Peking Union Medical College, Beijing, China; ^2^ Institute of Medicinal Biotechnology, Chinese Academy of Medical Sciences & Peking Union Medical College, Beijing, China

**Keywords:** cirrhosis, inflammation, liver injury, neutralizing antibody, p62

## Abstract

Interleukin-17A has been identified as a driver of hepatic stellate cell activation and plays a critical role in the pathogenesis of hepatic fibrosis. However, the underlining fibrosis-promoting mechanism of IL-17A is far from understood. Here we aimed to define whether hepatocytes directly respond to IL-17A stimulation and are associated with the development of hepatic fibrosis. The functional significance of IL-17A was evaluated in bile duct ligation (BDL) or thioacetamide (TAA) injection-induced mouse models of hepatic fibrosis. Human cirrhosis and control tissues were obtained from the patients with cirrhosis who received an open surgical repair process. Neutralizing IL-17A promoted the resolution of BDL or TAA-induced acute or chronic inflammation and fibrosis, resulted in a shift of the suppressive immune response in fibrotic liver toward a Th1-type immune response, and restored autophagy activity in both cholestatic and hepatotoxic liver injury induced fibrotic liver tissues, which was accompanied by a significant inhibition of STAT3 phosphorylation. Moreover, we found that IL-17A stimulated the concentration-and time-dependent phosphorylation of STAT3 in AML-12 liver cells. Blocking STAT3 with a specific inhibitor STATTIC or STAT3 siRNA protected from the IL-17A-induced autophagy suppression in AML-12 cells, indicating that STAT3 mediates IL-17A-suppressed autophagy. Administration of IL-10, which activated STAT3 and inhibited autophagy, reversed the therapeutic effect of IL-17A antagonism *in vivo*. Our study suggests that the IL-17A/STAT3 signaling pathway plays a crucial role in the pathogenesis of hepatic fibrosis through suppressing hepatocellular autophagy and that blocking this pathway may provide therapeutic benefits for the treatment of hepatic fibrosis.

## INTRODUCTION

Hepatic fibrosis is an excessive wound healing response that replaces the injured tissues by collagenous scars and causes organ failure finally. Indeed, hepatic fibrosis confers the structural basis for a variety of chronic liver diseases, including schistosomiasis, viral hepatitis, metabolic and alcoholic liver diseases [1, 2]. With persistent liver damage and inflammation, hepatic fibrosis may progress to cirrhosis, which is an advanced stage of hepatic fibrosis accompanied by a distortion of the liver vasculature and architecture, predisposing to liver failure and primary liver cancer [3]. Despite hepatic fibrosis especially cirrhosis is the major determinant of morbidity and mortality in patients with liver diseases and greater progression has been made in the understanding of pathogenesis of hepatic fibrosis in recent years [4], the effective antifibrotic agents to prevent or even reverse hepatic fibrosis is still an unconquered area for drug development [5, 6].

Th17 cells are an identified subset of effector CD4^+^ T cells, and IL-17A, the major cytokine released from these or other IL-17-producing cells, have been discovered to mediate autoimmunity and immune defense against pathogens [7]. A number of studies indicate that IL-17A is involved in the development of chronic fibroproliferative diseases. For instance, Baldeviano et al. reported that IL-17A promotes the development of dilated cardiomyopathy and blockade of IL-17A attenuates cardiac fibrosis and ameliorate ventricular function in experimental myocarditis [8]. We and others demonstrated that bleomycin induces pulmonary fibrosis in an IL-17A dependent manner [9, 10]. Lemmers et al. [11] and Ye et al. [12] respectively report that the IL-17A pathway involves in human alcoholic liver disease and hepatitis B. Meng et al. [13] and Tan et al. [14] indicate that IL-17A induces hepatic fibrosis via a direct activation of hepatic stellate cell (HSC) in patients with HBV and mouse models of liver fibrosis. Nonetheless, it remains unclear whether hepatocytes directly respond to IL-17A stimulation and are involved in the development of hepatic fibrosis. It would be interesting to elucidate other possible roles of IL-17A in hepatic injury induced fibrosis.

Autophagy maintains cellular homeostasis by facilitating the turnover of proteins and organelles involving lysosome. Accumulated evidence suggests that autophagy regulates apoptosis [15], restricts inflammation [16], and mediates fibrosis [17, 18]. We have recently found that blocking IL-17A attenuates experimental pulmonary fibrosis in mice, and we also demonstrated that IL-17A contributes into the development of pulmonary fibrosis by promoting TGF-β1-dependent collagen synthesis and TGF-β1-independent attenuation of autophagy in the fibrotic lung tissues [9]. Indeed, IL-17A promotes pulmonary fibrosis by attenuating autophagy via activating the PI3-kinase/GSK3b/Bcl-2 signaling cascade in lung epithelial cells [19]. Our preliminary data showed that fibrotic liver tissues expressed higher levels of IL-17A, IL-17R and its transcription factor retinoic acid-related orphan receptor-γt (RORγt) than control tissues. However, it is largely unknown whether increased IL-17A confers the inhibition of autophagy activity or whether regulation mediated by other molecules occurs downstream of IL-17A in the development of hepatic fibrosis. Our study indicates that antagonism of IL-17A attenuates liver fibrosis by restoring the STAT3-suppressed autophagy flux in the BDL-and TAA-induced fibrotic liver tissues. These data have been strengthened by the finding that administration of IL-10, a recently recognized inhibitor of autophagy through activation of STAT3 [20, 21], can reverse the beneficial effects of the IL-17A antagonism on experimental liver fibrosis. Our present study suggests that the IL-17A/STAT3 pathway is a potential therapeutic target for the treatment of fibroproliferative liver diseases.

## RESULTS

### Neutralization of IL-17A confers protection against hepatic fibrosis

**Figure 1:**
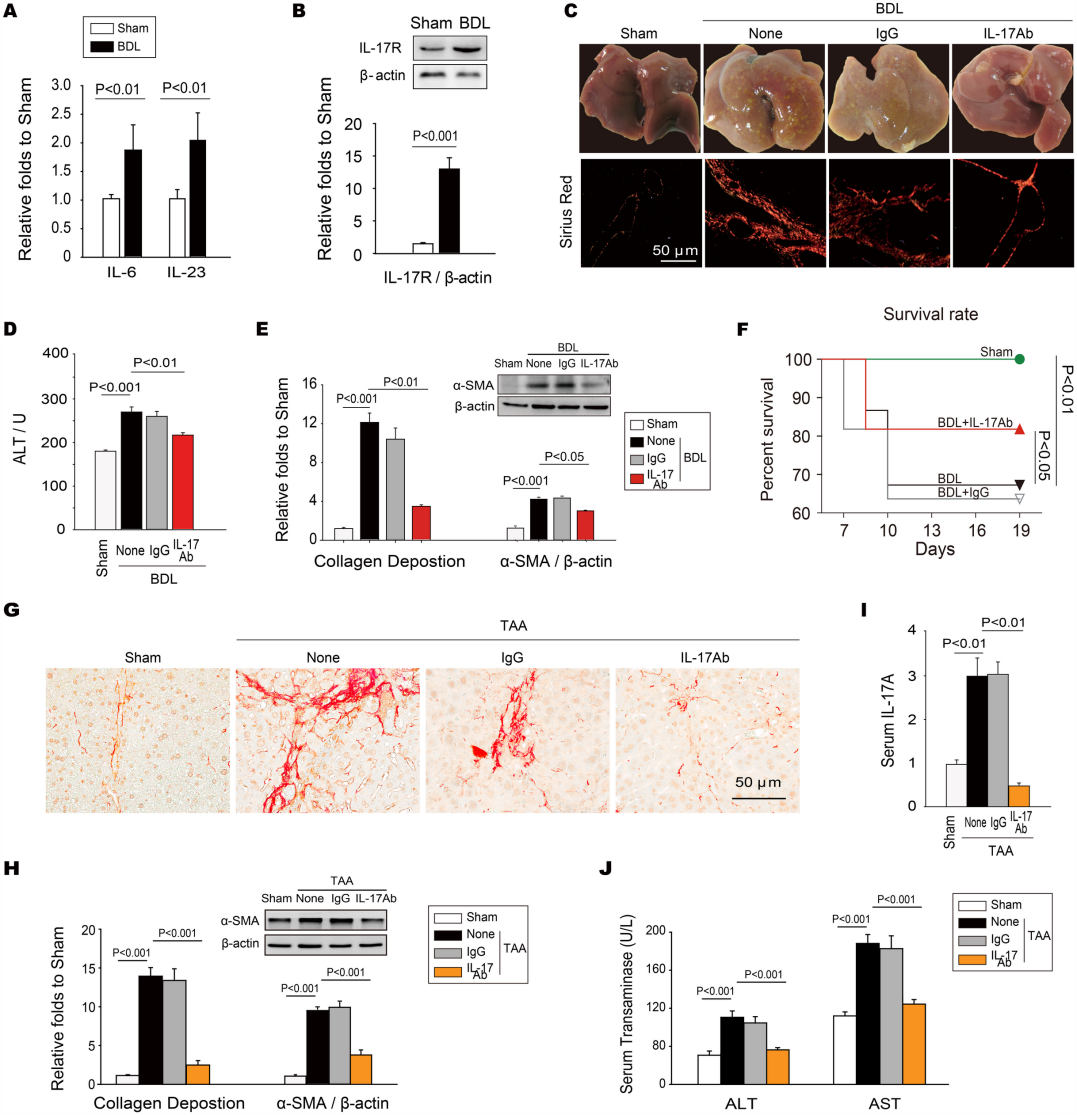
Neutralization of IL-17A inhibits the development of hepatic fibrosis **A**. Expressions of IL-6 and IL-23 were significantly increased in fibrotic liver tissue of the BDL-induced hepatic fibrosis. **B**. The IL-17R was also up-regulated in fibrotic liver tissues. **C**. Neutralizing IL-17A resolved BDL-induced cirrhosis. Data are morphology of the livers (top panels), as well as sirius red staining of representative photomicrographs of liver sections (bottom panels). **D**. Neutralization of IL-17A decreased the level of ALT in serum. **E**. Neutralizing IL-17A decreased the BDL-induced collagen deposition measured with sirius red staining, as well as the expression of α-SMA in hepatic tissues. **F**. Blocking IL-17A increased the survival rate of BDL-treated mice. The survival rate was analyzed by the Kaplan-Meier method (n=30/group). Neutralizing IL-17A also ameliorated TAA-induced chronic hepatic fibrosis. **G**. The representative photomicrographs of sirius red staining of the liver sections, **H**. the summary of collagen deposition and α-SMA expression, as well as the levels of IL-17A **I**., ALT and AST **J**. in serum indicated that neutralizing IL-17A could also ameliorate TAA-induced chronic hepatic fibrosis. Data are mean ± SEM (n=8 group^-1^ experiment^-1^) of three independent experiments.

We and others observed that both IL-17A and RORγt, the most important transcription factor of IL-17A, were up-regulated in BDL-induced fibrotic liver tissues [22]. Our preliminary experiment further showed that the expressions of IL-6 and IL-23 (Figure 1A), the close partners of IL-17A, as well as IL-17A receptor (IL-17R) were also increased in the fibrotic liver tissues (Figure 1B). Taken together, these data suggest that the IL-17A signaling pathway is activated in the fibrotic hepatic tissues. Because neutralizing IL-17A attenuates bleomycin-induced pulmonary fibrosis by promoting the resolution of inflammatory response [9], we therefore hypothesized that blocking the biological function of IL-17A might have therapeutic benefits against liver fibrosis. We found that BDL-stimulated significant fibrosis and collagen deposition in liver tissues; blockade of IL-17A conspicuously reduced hepatic fibrosis in BDL injured livers as indicated by an improved liver morphology (Figure 1C) and function (Figure 1D), decreased deposition of collagen and expression of α-smooth muscle actin (α-SMA) (Figure 1E). Moreover, the survival rate of the anti-IL-17A-treated group was markedly increased in comparison with that of the BDL-treated group (Figure 1F). Using TAA-induced chronic hepatic fibrosis of mouse models, we further found that neutralization of IL-17A not only decreased deposition of collagen and expression of α-SMA (Figure 1G and 1H), but also reduced the serum levels of IL-17A, ALT, and AST (Figure 1I and 1J) in TAA-treated mice. These findings suggest that IL-17A plays a crucial role in the pathogenesis of hepatic fibrosis and blocking IL-17A has a therapeutic efficacy against liver fibrosis.

### IL-17A antagonism reverses BDL and TAA-induced hepatic inflammation

**Figure 2:**
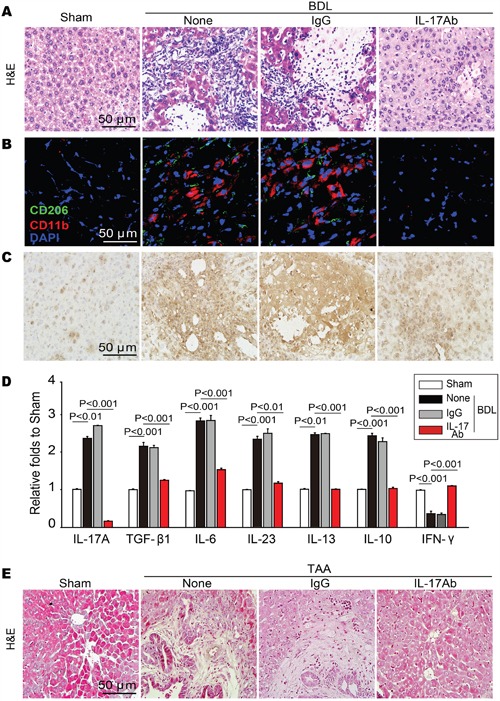
Neutralization of IL-17A resolved BDL or TAA-induced acute or chronic inflammation in hepatic tissue **A**. Neutralizing IL-17A repressed the BDL-induced recruitment of inflammatory cells as indicated by H&E staining of the liver sections, **B**. specially decreased the infiltration of M2-type macrophage (CD11b^+^CD206^+^). **C**. The representative photomicrographs of liver-infiltrating Th17 cells, which was assessed by immunohistochemistry. **D**. Blocking IL-17A attenuated the increase in the levels of IL-17A production associated Th2 and Treg cytokines but up-regulated the level of Th1 cytokine. The contents of IL-17A, TGF-β1, IL-6, IL-23, IL-13, IL-10 and IFN-γ in liver tissue homogenates were detected by ELISA analysis. **E**. H&E staining of the liver sections indicated that neutralization of IL-17A resolved the TAA-induced chronic inflammation. Data are mean ± SEM (n=8 group^-1^ experiment^-1^) of three independent experiments.

Because inflammatory response often precedes tissue fibrosis, the impacts of targeting IL-17A on the inflammatory responses were examined in fibrotic liver tissue. We found that the BDL-induced mechanical injury caused persistent acute inflammation by promoting the recruitment and infiltration of inflammatory cells in the lesion and the surrounding liver tissue; blocking IL-17A suppressed the recruitment of inflammatory cells (Figure 2A), specially decreased the infiltration of macrophage, among which M2-type macrophages were predominantly observed in the hepatic fibrotic tissues (Figure 2B). The number of Th17 cells and the level of IL-17A decreased for granted after blocking IL-17A in BDL-induced fibrotic tissues (Figure 2C and 2D). Previous work indicated that the polarized T Helper cells and the cytokines secreted by these cells can determine the development and progression of tissue fibrosis [23, 24], we examined the polarized responses of T cells and the cytokines in the fibrotic liver tissue. We found that IL-17A antagonism suppressed the high expression of TGF-β1, IL-6, IL-23, IL-13 and IL-10 in fibrotic hepatic tissues, but up-regulated the level of IFN-γ (Figure 2D). Blocking IL-17A exerted similar effect on recruitment and infiltration of inflammatory cells in the liver tissues from TAA-treated mice (Figure 2E). These data suggest that treatment with anti-17A Ab shifts the Th2-and Th17-polarized responses toward to a Th1-polarized response in injured hepatic tissues.

### IL-17A antagonism restores BDL and TAA-induced autophagy activity

**Figure 3:**
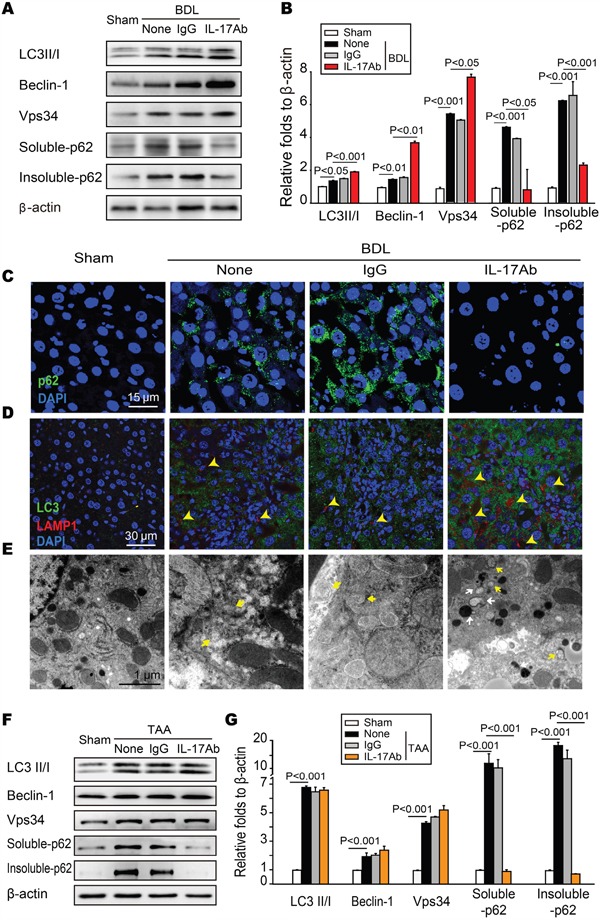
Neutralization of IL-17A restored BDL or TAA-induced autophagy suppression in hepatic tissue **A** and **B**. Neutralization of IL-17A regulated the expression of autophagy-associated proteins in BDL-treated livers. The livers were homogenated and the expression of LC3II/I, Beclin-1, Vps34, soluble-and insoluble-p62 was detected with Western blotting. Data are representative immunoblots and the ratios of the indicated protein to β-actin of three independent experiments. **C**. Blocking IL-17A enhanced the degradation of p62 in liver from BDL-treated mice, which was detected with confocal microscopy. **D**. Autolysosomes were evaluated with the co-localization of LC3 and LAMP-1 using confocal microscopy. Neutralizing IL-17A increased the numbers of autolysosomes (yellow arrows) in liver tissue. **E**. Blocking IL-17A increased the number of autophagosomes (Avi) and autolysosomes (Avd) in hepatocytes from BDL-treated mice. Data are representative images of electron microscopic analysis of three independent assays. Yellow arrows denote Avi; White arrows indicate Avd. **F-G** The expression of LC3II/I, Beclin-1, Vps34, soluble-and insoluble-p62 in TAA-induced liver hepatic tissue was analyzed with Western blotting. Representative immunoblots and the ratio of the level of indicated protein to β-actin from three independent experiments are presented.

Our recent research has shown that IL-17A attenuates the starvation-induced autophagy in the alveolar epithelial cells and that there is impaired autophagy in fibrotic lung tissues [9]. Thus, we wanted to know whether there was a similar change in fibrotic liver tissues and to determine how IL-17A modulated the autophagy activity. We found that the expressions of autophagy-associated proteins were enhanced after neutralizing IL-17A. Microtubule associated protein light chain 3 (LC3), a unique molecular marker of autophagysome, is widely recognized as an early mark of autophagy activation by its conversion of cytosolic form LC3-I to membrane-bound lapidated form LC3-II. The ratio of LC3-II/LC3-I was increased in fibrotic liver tissues; however, blocking IL-17A further enhanced this ratio. We then detected Beclin-1 and Vps34, two important signal proteins in autophagy, and expression of both was up-regulated; blocking IL-17A further increased their expressions (Figure 3A and 3B). As a marker of autophagy flux, the “cargo” protein p62, also called sequestosome 1 (SQSTM1), is involved in the trafficking of intracellular aggregates or unfolding proteins to the degradation pathway [25]. We found that p62 aggregation, as indicated by the numbers of p62-positive punctuate dots, showed a significant increase in BDL-injured liver tissues of untreated and IgG-treated mice compared with that in the liver tissues of sham and IL-17A Ab-treated mice (Figure 3C). Indeed, both of soluble-and insoluble-p62 were increased in the liver tissues of untreated or IgG-treated mice compared with that in the liver tissues of sham and IL-17A Ab-treated mice (Figure 3A and 3B). Moreover, blocking IL-17A promoted the formation of autolysosome, as IL-17A antagonism enhanced the co-expression of LC3 and LAMP-1 (Figure 3D). Transmission electron microscopy (TEM) analysis confirmed that only a small amount of autolysosomes (degrading autophagic vacuoles) were observed in fibrotic tissues from BDL-injured livers of untreated and IgG-treated mice. When treated with IL-17A antibody, both autophagosomes (initial autophagic vacuoles) and autolysosomes were dramatically increased in the fibrotic liver tissues (Figure 3E). Similar results of autophagy signals were obtained for TAA-treated mice (Figure 3F and 3G). These data indicate that there is a defective in autophagic flux in BDL or TAA-injured livers of untreated and IgG-treated mice but IL-17A antagonism reverses this impairment.

### IL-17A inhibits autophagy by activating STAT3

**Figure 4:**
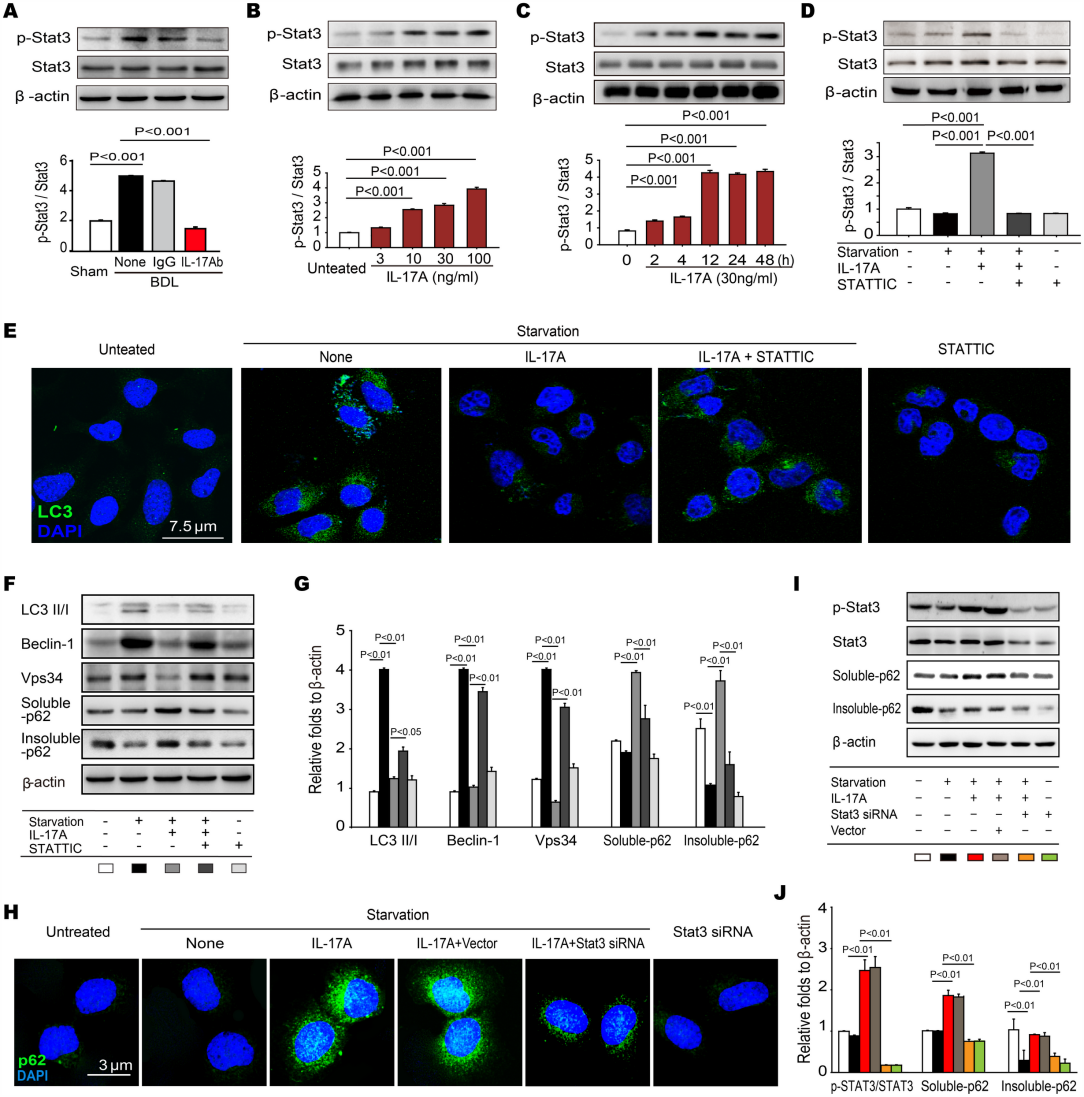
IL-17A inhibits autophagy by activating STAT3 **A**. The representative images of Western blots and the summary ratio of p-STAT3 to STAT3 in hepatic tissues. **B** and **C**. IL-17A stimulated the concentration-and time-dependent phosphorylation of STAT3 in AML-12 cells. The AML-12 cells were treated with the indicated concentrations of IL-17A for 24 hours or the cells were treated with 30 ng ml^-1^ of IL-17A for the indicated times, and the level of p-STAT3 and STAT3 was detected with Western blot assay. **D**. AML-12 cells were starved for 2 hours and treated with or without the indicated concentrations of IL-17A or STATTIC. The summary ratio of p-STAT3 to STAT3 was detected with Western blotting. **E**. Confocal analysis of LC3 expression in AML-12 cells. (F-G) STATTIC protected IL-17A (30 ng ml^-1^) from the inhibition of the starvation-induced autophagy in AML-12 cells. The expression of autophagy-associated proteins was detected with Western blotting. **H**. Silencing STAT3 prevented IL-17A-suppersed autophagy. STAT3 gene in AML-12 cells were silenced by STAT3 siRNA or vector and starved for 2 hours and treated with or without the indicated concentrations of IL-17A (30 ng ml^-1^). The aggregation of p62 was detected with confocal microscopy. **I-J** The expression of p-STAT3, STAT3, soluble-and insoluble-p62 in AML-12 cells was analyzed with Western blotting. Data are mean ± SEM of three independent assays.

**Figure 5:**
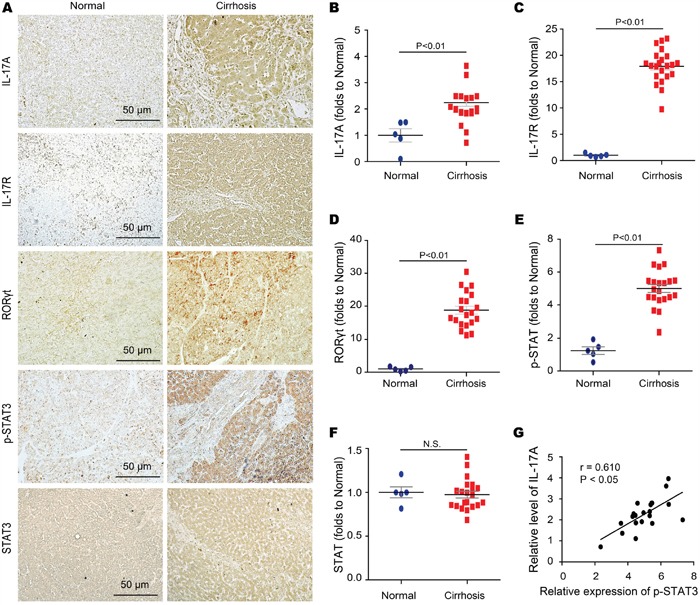
STAT3 phosphorylation correlates with the activated IL-17A signaling in human fibrotic liver tissues **A-F**. Expression of IL-17A IL-17R, RORγt, p-STAT3, and STAT3 were detected with immunohistologic staining in human cirrhosis and control liver tissues with quantized analyses of clinical samples. Data are representative of stained normal and cirrhosis liver tissues (left) with quantized analyses of paired clinical samples (right). **G**. Scatter plots showing the correlation of the STAT3 phosphorylation with the level of IL-17A in human cirrhosis tissues. Data are mean ± SEM and representative of 3 independent assays with identical results.

Although our work indicates that IL-17A signaling is responsible for the impaired autophagy both in fibrotic lung and liver tissues, the mechanism of IL-17A repressing autophagy is still unclear. Shen et al. reported that inhibition of STAT3 stimulates autophagy *in vitro* and *in vivo* but overexpression of STAT3 variants, encompassing wild-type, nonphosphorylatable, and extranuclear STAT3, inhibits starvation-induced autophagy [20]. Interestingly, we found that STAT3 was activated and anti-IL-17A Ab treatment protected from that in fibrotic liver tissues (Figure 4A), which was consistent with our previous observation that direct activation of autophagy caused a significant inhibition of STAT3 activity [21]. Indeed, IL-17A stimulated the concentration-and time-dependent phosphorylation of STAT3 in AML-12 cells (Figure 4B and 4C). To determine whether STAT3 activation mediated the IL-17A-suppressed autophagy, starvation-induced AML-12 cells were treated with IL-17A and the STAT3 antagonist STATTIC. Compared to starvation group, treatment with IL-17A resulted in 3.2-fold increase of p-STAT3/STAT3, which was protected from STATTIC treatment (Figure 4D). IL-17A treatment significantly inhibited the starvation-induced LC3 foci but IL-17A plus STATTIC treatment protected against the inhibition of starvation-induced LC3 foci (Figure 4E). The ratio of LC3-II/LC3-I and the expressions of signal proteins Beclin-1 and Vps34 were significantly decreased; both of soluble-and insoluble-p62 were accumulated with the administration of IL-17A. However, IL-17A and STATTIC reversed the expressions of these proteins (Figure 4F and 4G). Using siRNA-mediated depletion of STAT3, we found that IL-17A caused p62 accumulation in starvation-induced AML-12 cells whereas silencing STAT3 suppressed the IL-17A-induced p62 accumulation (Figure 4H, 4I and 4J). Using human liver tissue microarrays with cirrhosis, we further examined the expression levels of IL-17A, IL-17R, RORγt, STAT3 and p-STAT3 in 5 samples of normal liver tissue and in 22 samples of cirrhosis tissue by immune-histochemical staining. Higher expressions of IL-17A, IL-17R, RORγt and p-STAT3 were detected in cirrhosis tissues than that in normal liver tissues (Figure 5A–5E), whereas the level of STAT3 expression had no difference in cirrhosis or normal liver tissues (Figure 5A and 5F). Moreover, a positive correlation was observed between IL-17A and p-STAT3 levels in these cirrhosis tissues (Figure 5G). Altogether, these findings indicate that the phosphorylation of STAT3 mediates the IL-17A-suppressed autophagy of hepatocytes, which is involved in the development of hepatic fibrosis.

### IL-10 reverses the antifibrotic effect of IL-17A blockade via inhibiting autophagy

**Figure 6:**
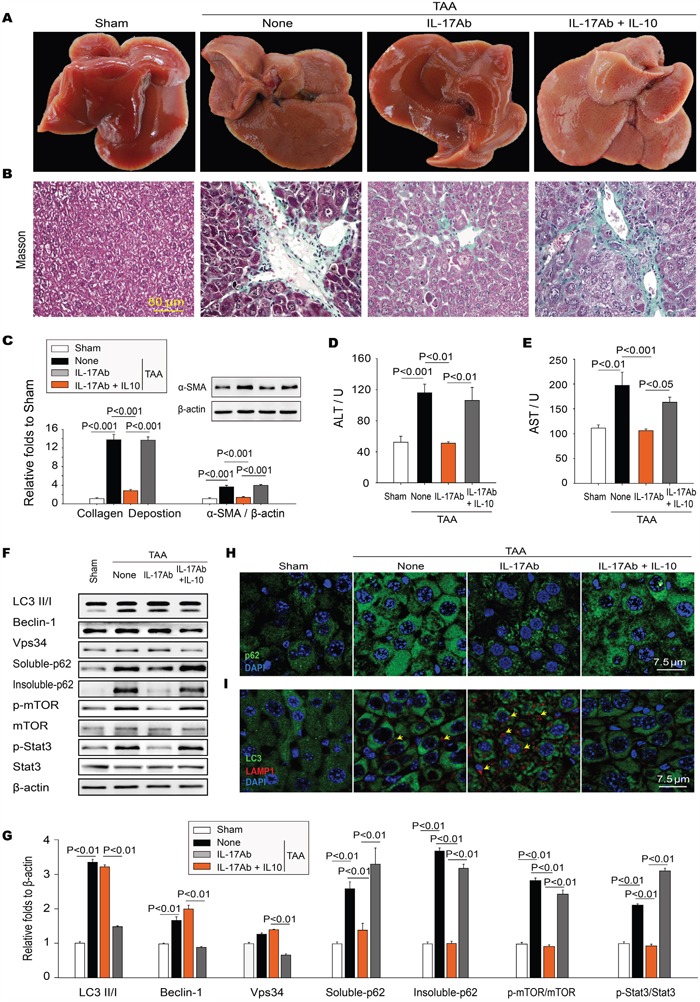
IL-10 reverses the anti-fibrotic effect of IL-17A antagonism in TAA-induced hepatic fibrosis **A**. Morphology of the livers. **B**. Masson's trichrome staining of representative photomicrographs of liver sections. **C**. Neutralizing IL-17A decreased the TAA-induced collagen deposition measured with Masson's trichrome staining as well as the expression of α-SMA in hepatic tissues, which were significantly reversed by IL-10. **D** and **E**. IL-10 prevented the IL-17A blockade-mediated decreased of the levels of ALT and AST in serum. IL-10 activated STAT3 impairing autophagy activity. **F** and **G**. The expression of LC3II/I, Beclin-1, Vps34, p-mTOR, mTOR, p-STAT3, STAT3, soluble-and insoluble-p62 in TAA-induced liver hepatic tissue was analyzed with Western blotting. Representative immunoblots and the ratio of the level of indicated protein to β-actin are presented. IL-10 increased aggregation of p62 **H**. and decreased the numbers of autolysosomes (I) in the IL-17A Ab treated livers detected with confocal microscopy. Autolysosomes are indicated by the coexpression of LC3 and LAMP-1 (yellow arrows). Data are mean ± SEM (n=8 group^-1^ experiment^-1^) of three independent experiments.

Because the neutralization of IL-17A restored autophagy activity and attenuated the hepatic fibrosis, and IL-17A inhibited autophagy by phosphorylating STAT3, we examined whether inhibiting autophagy by activating STAT3 could reverse the antifibrotic effect of IL-17A-neutralizing Ab. Treg cytokine IL-10, which has been coupled with the activation of JAK-STAT1 or STAT3 signaling cascade [26, 27] and found high expression in liver fibrotic tissues, was used to reverse therapeutic effect of IL-17A antibody in TAA-induced hepatic fibrosis mice. Indeed, IL-10 prevented the IL-17A blockade-mediated improvement of liver morphology (Figure 6A), reduction of collagen deposition (Figure 6B and 6C) and expression of a-SMA (Figure 6C), as well as liver function (Figure 6D and 6E), in fibrotic liver tissue. We also found that IL-10 hindered the IL-17A blockade-mediated restoration of autophagy activity. Treatment with IL-10 decreased the ratio of LC3-II/LC3-I, the expression of Beclin-1 and Vps34, which were increased in anti-IL-17A treatment group. Moreover, the mammalian target of rapamycin (mTOR), a key inhibitor of autophagy, was markedly increased in the liver tissues of IL-17Ab and IL-10 treated mice (Figure 6F and 6G). The protein p62 was reduced with IL-17A-neutralizing Ab treatment, whereas IL-10 treatment aggravated the accumulation of p62 again (Figure 6F, 6G and 6H), as well as preventing the IL-17A-neutralizing Ab promoted the formation of autolysosome (Figure 6I). Taken together, these findings indicate that IL-10/STAT3 activation mediates IL-17A-suppressed autophagy and participates in the development of hepatic fibrosis.

## DISCUSSION

Recent studies have demonstrated that IL-17A plays a crucial role in the development and progression of tissues fibrosis including hepatic fibrosis [8, 9, 11]. In this study, we find that the expressions of IL-17A and IL-17A-related molecules as well as components of the IL-17A signaling pathway are increased in the fibrotic liver tissues caused by cholestatic liver injury. Blocking IL-17A activity shows a therapeutic efficacy against both cholestatic and hepatotoxic hepatic fibrosis and results in a shift from the suppressive immune response toward a Th1 response in the fibrotic liver. Importantly, we found that the antifibrotic effects of IL-17A antagonism were due to the restoration of IL-17A-suppressed autophagy via phosphorylation of transcriptional factor STAT3. Indeed, both STAT3 inhibitor STATTIC and STAT3 siRNA protected from the IL-17A-induced autophagy suppression. STAT3 activation is critical for IL-10 production [28], especially the induction of IL-10 in Th17 cells by a combination of TGF-β1/IL-6 was also found to require STAT3 activation [29, 30]. Our study demonstrated that activation of STAT3 and inhibition of autophagy with IL-10 reversed the antifibrotic effects of IL-17A antagonism.

Meng et al. demonstrated that IL-17A directly activates HSCs and induces hepatic fibrosis in a STAT3-dependent manner [13]. Activation of STAT3 and its downstream signal activity have been implicated in the control of hepatic fibrogenesis, which can be initiated or mediated by a variety of inflammatory cytokines, growth factors, and hormones [31]. Although Meng et al. had extensively dissected several types of IL-17-responsive cells contributing to the pathogenesis of liver fibrogenesis, several questions, particularly how IL-17A induces hepatic fibrosis, remain need to be addressed [32]. Indeed, our current study has further explored the mechanism of IL-17A in the regulation of hepatic fibrosis and provides insights for answering these questions. For instance, Meng et al.'s studies revealed that IL-17A exacerbate hepatic fibrosis by focus on how inflammatory cells respond to the changes of IL-17A signaling in response to liver injury; whereas our study confirmed that IL-17A promoted hepatic fibrogenesis by focus on how the intracellular autophagy activity is regulated by the activation of IL-17A signaling in response to liver injury. Also, we determine the subpopulation of liver-infiltrating macrophages in BDL-induced fibrotic liver tissue and find that M2 type macrophages are predominantly infiltrated in fibrotic liver tissues and the treatment with IL-17A-neutralizing Ab decreases the infiltration of M2 macrophages. Moreover, our work indicates that HSCs are not only cell population to contribute to hepatic fibrosis in response to IL-17A via STAT3 activation as shown by Meng et al's work. Indeed, we find that hepatocytes are also participated in liver fibrogenesis in response to IL-17A activation of STAT3 signaling.

Our previous work indicates that Th17 cells and cytokine IL-17A participate in the development of pulmonary fibrosis through suppressing autophagy [9]. IL-17A stimulates the production of Th2 and Treg cytokines such as TGF-β1 and IL-10 to create a suppressive immune microenvironment in fibrotic lung tissue, which may interfere with autophagic clearance of inflammatory inducers, which may sustain chronic inflammation and promote the progression of pulmonary fibrosis. In current study, we further find that IL-17A inhibits autophagy via the activation of STAT3, which may hinder the degradation of collagen and the clearance of injured tissue debris and interfere with the resolution of hepatic fibrosis. It seems that IL-17A induces tissue fibrosis in different organs by utilizing different mechanisms. Indeed, we find that IL-17A promotes pulmonary fibrosis through the activation of PIK3CA to interrupt the GSK3B-mediated degradation of BCL2 and suppress autophagy in lung epithelial cells [19], whereas IL-17A induces hepatic fibrosis through activating the STAT3 pathway and interfering with the p62 mediated autophagy flux in fibrotic liver tissue. These results indicate that IL-17A produces the profibrotic roles in different organs through a universal mechanism-suppressing intracellular autophagy activity but with different regulatory details.

There is always a doubt whether IL-10 could be used for anti-fibrotic clinical therapy, since different animal models of acute or chronic liver injury reveal quite dissimilar effects of IL-10 on fibrosis [23]. Several studies inhibited IL-10 by genetic way, and found IL-10 depletion resulting in liver injury or fibrosis exacerbation [33, 34]. As we know, IL-10 is an important anti-inflammatory cytokine that suppresses the expression of proinflammatory genes, using genetic way to completely deplete IL-10 would certainly aggravate the inflammation and fibrosis. However, from our studies, in the fibrotic tissue, the expression of IL-10 is so high that it would result in an immunosuppressive microenvironment, which is harmful for the improvement of fibrosis as we have mentioned before, and IL-10 indeed reversed the antifibrotic effects of IL-17A antagonism. In support of our findings, Lee et al. found that transgenic overexpression of IL-10 in the lung causes fibrosis [35]. With regard to these findings, anti-fibrotic therapy of IL-10 needs to be verified.

In summary, our current study not only indicates the IL-17A/STAT3 pathway playing a critical role in the pathogenesis of hepatic fibrosis, but also provides the proof of concept for this pathway acting as a potential therapeutic target against hepatic fibrosis.

## MATERIALS AND METHODS

### Materials

The mouse IL-17A protein was purchased from Peprotech (Rocky Hill, NJ). The neutralizing mouse IL-17A mAb was obtained from R&D Systems (Minneapolis, MN). Anti-mouse α-SMA, IL-17R, RORγt, p-STAT3, STAT3, LC3 II/I, Beclin-1, LAMP-1, Vps34, CD11b, CD206, and β-actin Abs were purchased from Cell Signaling Technology (Danvers, MA). Anti-mouse p62 Ab and thioacetamide (TAA) were purchased from Sigma (St. Louis, MO). Alexa Fluor 488 and 647 Abs were obtained from Invitrogen (San Diego, CA). The assay kits for Alanine aminotransferase (ALT) and aspartate aminotransferase (AST) were obtained from Nanjing Jiancheng Bioengineering Institute (Nanjing, China). The ELISA kits for IL-17A, TGF-β1, IL-6, IL-23, IL-13, IL-10 and IFN-γ were purchased from eBioscience (San Diego, CA). Other materials were purchased from commercial sources.

### Animal model of hepatic fibrosis

Male BALB/c mice (6-8 week old) were obtained from Vital River Laboratory Animal Technology (Beijing, China). All animal protocols conformed to the Guidelines for the Care and Use of Laboratory Animals prepared and approved by the Animal Care and Use Committee of Chinese Academy of Medical Sciences and Peking Union Medical College. The BDL model was generated following a previous report [36]. Mice were anesthetized with 45 mg kg^-1^ i.p. of pentobarbital and the abdominal cavity was opened from the abdominal midline. The common bile duct was ligated twice with 1-0 silk suture and the bile duct was cut between the two ligations. Sham-mice were only operated to laparotomy without BDL. To generate a TAA-induced model of hepatic fibrosis, mice were i.p. injected with TAA in PBS (200 mg kg^-1^) at 2 times week^-1^ for 12 weeks. The mice were i.v. injected with IL-17A-neutralizing or isotype-matched Ab (2 μg mouse^-1^) on days 0, 5 and 10 after BDL procedure or every 5 days from the 6th week after first TAA injection. At the end of experiments, the mice were sacrificed by excessive anesthesia for the collection of serum and livers. The livers were excised and fixed or frozen for morphological evaluation.

### Human cirrhosis tissue microarray

The paraffin-embedded human liver tissue microarray slides which contain 22 cases of cirrhosis and 5 cases of normal liver tissue specimens were purchased from US Biomax (Rockville, MD). The tissue microarray was detected by immunohistochemical analysis as previously described [37]. All protocols using human specimens were approved by the Institutional Review Board of Chinese Academy of Medical Sciences and Peking Union Medical College. Informed consent was obtained from all subjects. The study conforms to the principles outlined in the Declaration of Helsinki.

### Cell culture

The mouse hepatocytes (AML-12) were purchased from the ATCC (Manassas, VA), which were cultured in DMEM-Ham's Nutrient Mixture F12 (1:1; Hyclone) supplemented with 10% FBS and 100 mg ml^-1^ penicillin/streptomycin. The cells were passaged every 2 days.

### Histomorphology

The livers were fixed with 4% paraformaldehyde, and embedded in paraffin for histopathological examination. Liver tissue sections (5 μm thick) were stained with H&E, sirius red, or Masson's trichrome blue. The grade of hepatic inflammation and fibrosis was blindly assessed by a professional pathologist. The average integrated OD (IOD) of collagen deposition from 10 randomly chosen regions per tissue sample at a magnification of ×200 was calculated using Image-Pro Plus 5.1 image analysis software.

### Confocal assay

Standard protocols for immunofluorescence microscopy were used. AML-12 cells were planted on the coverslip-bottom dishes and treated with or without indicated agents. The cells were fixed with 4% paraformaldehyde for 1 h, and washed three times after fixing. The cells on coverslips or liver sections (5 μm thick) were stained with indicated primary Abs overnight at 4°C. The coverslips or sections were washed thrice, and then incubated with Alex488-or Alex647-conjugated secondary Abs (1:200) for 30 min. Nuclei were visualized by 4, 6-diamidino-2-phenylindole staining (DAPI). Images were obtained with a Leica SP2 confocal microscope (Leica Microsystems, Exton, PA) and analyzed using Leica confocal software. The autophagosomes were identified by LC3 dots, and the autophagolysosomes were identified by the coexpression of LC3 and LAMP-1.

### Transmission electron microscopy

Mouse tissues were fixed with 2.0% glutaraldehyde in 0.1M sodium cacodylate buffer, pH 7.4, and then post-fixed in 1% osmium tetroxide, dehydrated in ethanol and embedded in epon. Ultrathin sections of liver tissues were collected on formvar-coated grids and were stained with uranil acetate and lead citrate. The samples were examined with a HITACHI H600 Transmission Electron Microscope operated at 80 KV.

### ELISA

The concentrations of IL-17A, TGF-β1, IL-6, IL-23, IL-13, IL-10 and IFN-γ in liver tissue homogenates were detected by enzyme-linked immunosorbent assay (ELISA) using kits from eBioscience according to the manufacturer's instructions.

### ALT and AST assay

Blood samples were collected from the orbital sinus and incubated for 1 h at room temperature to allow clotting. Then the serum were collected by a centrifugation at 3000 RPM for 15 min and stored at -80°C until use. Serum alanine aminotransferase (ALT) and aspartate aminotransferase (AST) were assayed using an ARKRAY SPOTCHEM SP-4410 automatic dry chemistry analyzer.

### Western blotting

Protein samples were extracted from liver tissue or cultured AML-12 cells with procedures as described in detail elsewhere [38]. For insoluble fraction preparation, pellets were washed with RIPA and resuspended in 2% SDS Tris-buffer. Protein concentrations were determined using a BCA reagent. Equal amounts of protein were subject to SDS-PAGE. Binding of the primary antibody was detected by peroxidase-conjugated secondary antibodies and enhanced chemiluminescence, and bands were quantified with Amersham ECL Western Blotting Detection System (GE Healthcare).

### Statistical analysis

All results are represented as the mean ± SEM. Two-group comparisons were made by Student's t test as appropriate, while multiple comparisons among three or more groups were performed using one-way ANOVA, p<0.05 was considered statistically significant. The survival rates were analyzed using the Kaplan-Meier method. All statistics were analyzed using SPSS 17.0 software.
